# Determining the Requirements of Vulnerable Groups for Health Counseling and Optimizing the Evaluation of Health Consultations: Mixed Methods Study With the Use of AI

**DOI:** 10.2196/68888

**Published:** 2025-06-04

**Authors:** Annina Boehm-Fischer, Luzi M Beyer

**Affiliations:** 1 Alice-Salomon-Hochschule Berlin, Alice-Samolon-Platz 5, Berlin, 12627, Germany, 49 30939397 ext 720

**Keywords:** health counseling service, health service evaluation, immigrant health, mixed methods, online questionnaire, vulnerable populations, in-depth interviews

## Abstract

**Background:**

Evaluating health counseling services is crucial for ensuring their quality and effectiveness. However, this process is hampered by challenges such as language barriers and limited awareness of their needs and concerns.

**Objective:**

The studies aimed to enhance and digitize an existing paper-and-pencil evaluation form for a health counseling service while gaining insights into client needs and barriers. This effort intends to adapt a health care facility’s offerings to better meet client demands and implement a multilingual format for greater accessibility.

**Methods:**

The research team designed and conducted an in-depth interview study with clients of a health counseling service to gather new information. The insights regarding client demands, wishes, and social needs were used to revise and supplement the existing 1-page questionnaire (originally in German) for evaluating counseling sessions. Using artificial intelligence, the team transformed the new 3-page questionnaire into easy language with a Kunin smiley scale, translated it into 7 other languages, and created audio recordings for all questions in each language. The questionnaire was then programmed into an web-based tool, allowing data collection both on-site with tablets and through integration into the counseling service’s website. This digital format is now continuously used to adapt the counseling service to clients’ needs.

**Results:**

A total of 18 clients participated in the in-depth interviews, which were conducted in their native languages whenever possible and lasted between 8 and 30 minutes. The results indicated that many clients attending the counseling center are burdened by physical and mental health issues, with a significant portion of the assistance provided focused on helping clients complete various forms required by health insurance providers and medical professionals. Despite these challenges, clients expressed a high level of satisfaction with the health counseling services they received. The revised and supplemented web-based questionnaire has been completed by 41 clients. Evaluation results revealed that only 21 respondents (51%) filled out the questionnaire in the national language (German), while English and Arabic were the next most common choices, each used by 6 clients (15%). Findings regarding health burdens and the need for assistance were reaffirmed, highlighting that clients’ self-perception regarding their ability for self-help is notably low.

**Conclusions:**

Contrary to previous assumptions, it was found that client interests predominantly lie in receiving help with the excessive demands imposed by institutional forms and requirements rather than solely addressing health issues. Clients showed strong satisfaction with the advice received and emphasized the necessity for multilingual health counseling services and evaluations. There is a distinct need for support in completing forms for doctors and health insurance applications. In addition, many clients expressed a lack of confidence in managing health care processes independently in the future, underscoring the need for greater awareness of available resources and support networks.

## Introduction

The German health care system, despite being one of the most expensive globally, offers extensive services, high-quality standards, low access barriers, and income-independent health care [1]. In theory, this should ensure excellent care for all. However, in practice, barriers exist that limit access to health care services. Non-native speakers and individuals not born in Germany often lack information about health services and face language barriers [2,3]. Their utilization rates of health care services during illness are significantly lower compared with other European countries [4], and they struggle with understanding and processing health-related information [5]. In addition, preventive health services in Germany are less frequently used by elderly individuals, those living alone, and people requiring informational support [6,7]. A representative survey indicates that less than half of older adults in Germany (aged 75 years and older) have internet access, and of those, only about half (thus, about a quarter overall) use the internet to search for health-related topics [8]. A trend emerges in this demographic: lower education levels, smaller social networks, and reduced quality of life correlate with less internet usage. Furthermore, individuals with lower household incomes and educational levels are less likely to use digital health technologies in Germany [9]. As a result, vulnerable groups in Germany are just as dependent on high-quality health counseling services (HCSs) as in other countries [10-16].

Health counseling plays a critical role in improving public health, particularly for vulnerable groups, making the evaluation of these services essential for ensuring quality. Research on the effectiveness of HCS has been conducted for over 50 years [17]. Early studies primarily focused on procedural and outcome assessments with limited client involvement [18]. More recent evaluations have shifted toward a client-centered approach [19,20], acknowledging that actively involving clients in the design of health consultation assessments yields valuable insights that differ from those of health care professionals [21]. This participatory approach ensures that specific client needs are adequately focused on [22].

Qualitative research methods are particularly useful for capturing opinions, social needs, and desires. However, web-based qualitative studies present challenges regarding accessibility and engagement [23]. Therefore, an in-depth face-to-face interview study is recommended as an initial approach. This method is particularly suited for discussing health questions and interactions with the health care system [24].

For the revised version of the HCS evaluation form, an web-based questionnaire with a smiley response scale (Kunin scale) is effective. This format enables multilingual adaptation and audio-assisted completion, ensuring accessibility regardless of language barriers. It can be completed independently of time and location, reducing the risk of socially desirable responses [25], while allowing a greater flexibility in participation.

**Figure 1.**
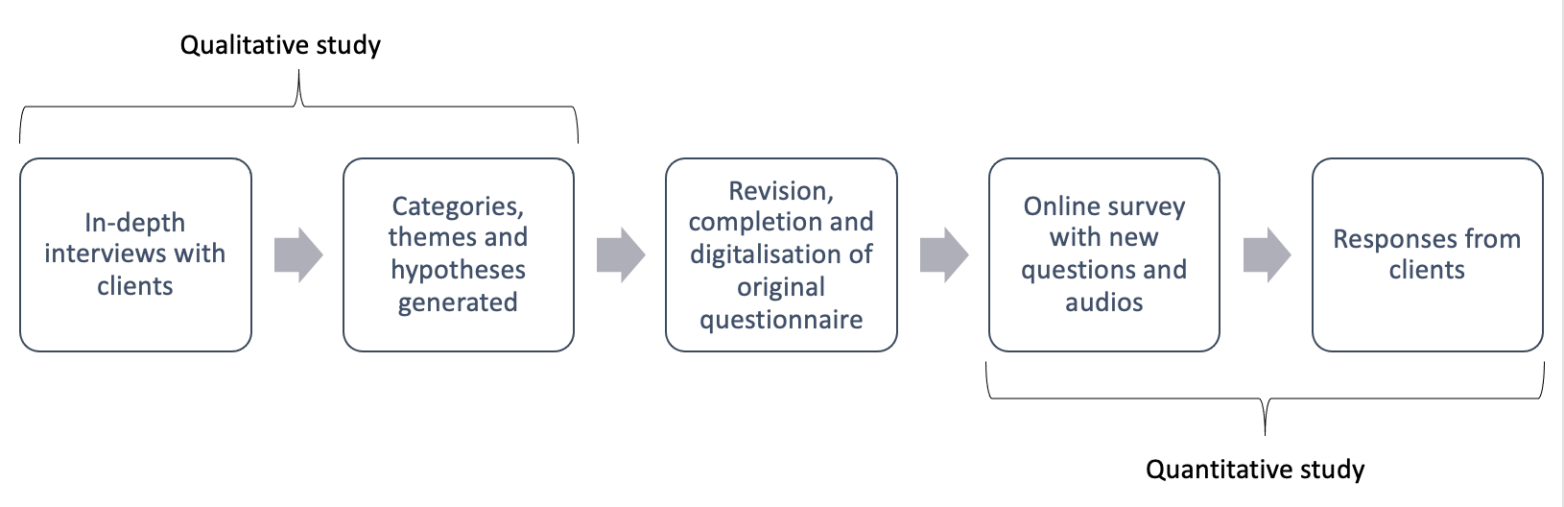
Flowchart of study design in study. The planned duration was 12 months (April 2023 to March 2024). Based on the interview study with clients of a health counseling service, Berlin (Germany), the existing German evaluation form was to be expanded, improved, and digitally implemented in several languages in order to better meet the needs of the clients. After the subsequent quantitative studies (and any necessary adjustments), the evaluation form should be handed over to the health counseling service.

With the aim of customizing the evaluation form and involving the clients, we conducted a 2-stage mixed methods study (see Figure 1).

The clients of the health care facility have diverse native languages, some are illiterate, and many have a combination of mental and physical impairments. To be able to address them, this study followed a mixed methods approach consisting of 2 phases: a qualitative in-depth interview survey and a subsequent quantitative web-based survey with the following research questions (see Textbox 1).

Textbox 1. **Research questions.**

Qualitative in-depth interview survey:

- What are typical consulting contents and types of services provided?
- What quality characteristics are mentioned by users in relation to the HCS (eg, length of sessions, perceived professionalism, feeling supported)?
- What quality characteristics do users mention in relation to the staff (eg, goal-oriented, understanding)?
- What additional needs are mentioned?
- Is the service perceived as recommendable?

Quantitative web-based survey study:

- Which languages are preferred when completing the form?
- From whom are advice seekers sent and what are the reasons for using the advice center?
- What is the subjective perception of the users with regard to the quality characteristics determined by users in the qualitative study in relation to the service and staff?

## Methods

### Overview

A mixed methods approach was selected to gain an in-depth understanding of clients’ challenges (qualitative), which served as the foundation for developing relevant questions and response categories for the questionnaire (quantitative), ensuring both relevance and broad applicability.

### Ethical Considerations

Before the study was conducted, an internal ethical review was conducted at the Alice Salomon University of Applied Sciences by an independent research ethics committee, based on the guidelines of the German Ethics Council. There was a consensus discussion regarding the ethics review and implementation guidelines with the staff and supervisors of the HCS. It was determined that the interviews with clients would only address topics that are also discussed during the counseling sessions. It was agreed that all interviews would take place in the counseling rooms and that a staff member would always be present in other rooms to provide support as needed. The interviews would be fully anonymized to protect privacy and confidentiality, no video recordings would be made, and the audio would not be made public; only the transcripts would be shared. Under these conditions, the consensus discussion concluded that there were no concerns. The result of the ethical evaluation was that the research ethics criteria for the study were satisfactorily met and the study may be conducted under the conditions.

Informed consent was obtained from all interviewees, which also permitted secondary analysis. No demographic information was requested, and any personal information was anonymized during transcription (eg, when discussing individual staff members, names were replaced with XXX).

The interviewees did not receive any financial or other compensation for their participation in the interviews.

### Study 1: Qualitative In-Depth Interview Study

#### Interview Development and Procedure

Before the qualitative study, a workshop was held with the HCS team to exchange thoughts regarding objectives, desires, and methodologies. During this workshop, the original evaluation questionnaire, which comprised 7 questions, was reviewed.

A literature search on evaluation questionnaires used in health counseling was conducted, and a draft for the interview was developed. This draft was subsequently refined in a workshop, during which the questions were organized. The final interview composed of 8 primary questions and an additional 14 probing questions aimed at encouraging participants to elaborate on their responses.

The final interview guide included 8 primary questions and 14 probing questions. Interviews were conducted in German, English, or in participants’ native languages to ensure clarity and accuracy. Interviewers were multilingual, allowing direct communication or real-time translation when necessary. Interviews were recorded as MP3 files and transcribed using artificial intelligence (AI)–based transcription software (noScribe). This software was selected due to its multilingual support and its capacity to run locally on a personal computer, thereby circumventing the need to upload audio files to the internet. Following the transcription process, the texts were compared with the audio files by native speakers of the respective languages. Necessary corrections were made, after which the responses were translated into German using an AI-assisted translation tool (DeepL). The translated responses were subjected to a thorough review to ensure content accuracy.

Subsequently, all responses to the individual questions were analyzed separately in a workshop setting, and where feasible, an inductive category system was developed according to Mayring’s methodology [26].

For the responses to the first question, “How long have you been receiving counseling?” and its corresponding probing questions (“How often have you attended counseling since then?” and “What was your experience in scheduling an appointment?“), no category system was established due to the nature of the responses. For the second question, “What health topics have you sought counseling for?” the response categories were deductively derived from the answers and included physical health, mental health, other topics, and categories related to filling out applications, counseling, and referrals.

Regarding the third question, “Was the time sufficient?” and the two probing questions (“Were you able to discuss all health issues?” and “Did you feel well supported?”), no category system was created, as the responses were too homogenous.

For questions 4 (“How did you experience the counseling?“) and 5 (“How did you perceive the staff?”) along with their corresponding probing questions, a unified category system was developed, since many participants did not differentiate between the counseling service and the personnel involved. The categories established were: statements regarding atmosphere, outcome-orientedness and objective-orientedness of the counselors, counseling demeanor and conduct, expressed trust and security, and expressed difficulties.

For the sixth question (“How do you feel after the consultation?”) and the first exploratory question (“To what extent did the health advice help?”), a category system was developed with the categories “Mentioned emotions,” “Statements about stress” and “Other.”

For the second probing question regarding the sixth inquiry (“In what areas do you seek further support?”), a category system was deductively created based on the responses, including categories such as physical and mental health, assistance with applications, case management services, language acquisition, housing search, job search, and networking.

For the seventh question (“Will you be able to cope with issues independently in the future?”), as well as its corresponding probing question (“Do you now know where and how to seek further help?”) an inductive category system was developed with the categories “Need for help due to chronic mental/physical health problems,” “Overburdened with structures in the healthcare system,” “Language barriers (eg, at the doctor’s or hospital),” “Barriers due to lack of reading/spelling skills,” “No other help (caregiver, other advice center) known” and “Other.” No category system was developed for the eighth question (“Would you recommend health counseling?”) and the 3 following probing questions (“How well do you feel supported?,” “What further health counseling would you like?,” and “Is there anything else you would like to tell us?”), in order to do justice to breadth, individuality, and nonspecificity of the answers.

#### Data Collection

The primary objective of the recruitment process for the interview study was to enlist a total of 12 individuals identified as “clients” (individuals who had attended counseling in person at least twice, for whom contact information was available, and who expressed a desire for further consultations), along with 6 individuals categorized under “case management” (those requiring on-site consultations coupled with assistance in navigating administrative and medical appointments, which may include home visits), and 6 individuals seeking walk-in consultations who were attending for the first time and preferred to remain anonymous. This recruitment goal was established to reflect the approximate distribution of service recipients within the counseling framework.

For reasons of practicability, the recruitment procedure was as follows: The external interviewers told the health counseling staff when they would have time to conduct interviews (July 3‐7, 2023). They then asked already scheduled clients (clients and case management) whether they could come earlier or still have time for an interview after their appointment (telephone contact). Through this process, 12 clients and the 6 case management participants were contacted and all those contacted agreed to participate. In the end, 10 clients and the 6 case management participants took part in the planned sessions. While the interviewers were on site, all walk-in clients were asked if they had spontaneous time for an interview. Of the 6 people approached who wanted to take advantage of counseling, 2 agreed to participate, while the remaining 4 declined. The inclusion criterion was that they had to have already received counseling at least once. All potential participants received information and two written informed consent forms before the interview, one of which they had to sign and return. However, no employees were present during the interviews in order to avoid potential conflicts of interest on the part of the interviewees.

After the audio recordings were made (before coding), 1 individual categorized under case management was excluded from the analysis, as this participant provided extensive narratives but failed to respond to any of the posed questions. Subsequently, another case management participant was added to the sample as a compensatory inclusion.

Thus, the final sample for the interview study comprised 10 clients, 6 case management participants, and 2 walk-in consultation seekers (n=18 of 24 asked persons, 75% response rate). The data collection, coding, and the data analysis was completed in July 2023. A total of 10 people (the authors and 8 students) were involved in the data collection, coding, and analysis.

#### Data Analysis

After coding the transcripts of the interview study with QCAmap [26,27], a qualitative content analysis according to Mayring [26] was carried out.

For the responses to the first question, “How long have you been receiving counseling?” and its corresponding probing questions (“How often have you attended counseling since then?” and “What was your experience in scheduling an appointment?“), no category system was established due to the nature of the responses. For the second question, “What health topics have you sought counseling for?” the response categories were deductively derived from the answers and included physical health, mental health, other topics, and categories related to filling out applications, counseling, and referrals.

Further analysis included questions on session duration, counseling experience, perception of staff, and additional support needs, ensuring that both homogeneous and diverse responses were categorized effectively. These categories were then refined through collaborative coding sessions before finalizing the thematic framework.

#### Data Exclusion

One interview from the in-depth interview study was completely excluded from analysis due to the absence of any substantively evaluable responses to the questions posed. Among the interviewees, there were also 2 people who were unable to answer some of the questions even after repeated questioning, rephrasing, and shortening, but spoke at times in a very repetitive, over-excited and unfocused manner. Some responses from these interviews were excluded.

### Study 2: Quantitative Web-Based Survey Study

The insights gained from the qualitative interview study were used to develop a structured questionnaire.

#### Questionnaire Development and Implementation

Before the quantitative study, a second workshop was conducted with the HCS team to discuss the outcomes of the interview study and to coordinate forthcoming actions. The responses from the interview study, along with the original paper-and-pencil questionnaire, were critically examined. Subsequently, an additional literature search was carried out.

The questionnaire was developed in German in August, after which it was initially transformed into a simplified language format using artificial intelligence (ChatGPT). Following this transformation, the content was translated into English using an AI-based tool (DeepL), since translations from English into other languages (eg, Russian, Arabic) tend to yield better results than translations from German. In September, the English questionnaire was translated into 6 additional languages (Arabic, French, Russian, Romanian, Spanish, and Turkish), and it was cross-checked by native speakers. This process resulted in the questionnaire being available in a total of 8 languages.

**Figure 2.**
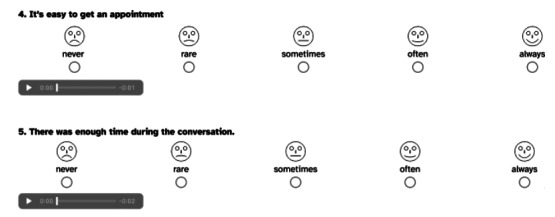
The 2 questions from the English questionnaire of the quantitative study in which there were 8 content-identical language translations. Under each question is a read-aloud option and all answers are as smiley faces so that questions can be answered without reading skills.

Subsequently, audio files for all questions in all languages were generated using AI (NVIDIA Jarvis), resulting in a total of 146 questions (18 questions multiplied by 8 languages plus language selection and consent to data processing). The audio recordings were reviewed by native speakers in October, and edits were made as necessary. The finalized audio files, along with the questions, were then programmed into a web-based tool (SoSciSurvey, see Figure 2). Functionality tests were subsequently conducted to ensure the operability of the survey.

**Figure 3.**
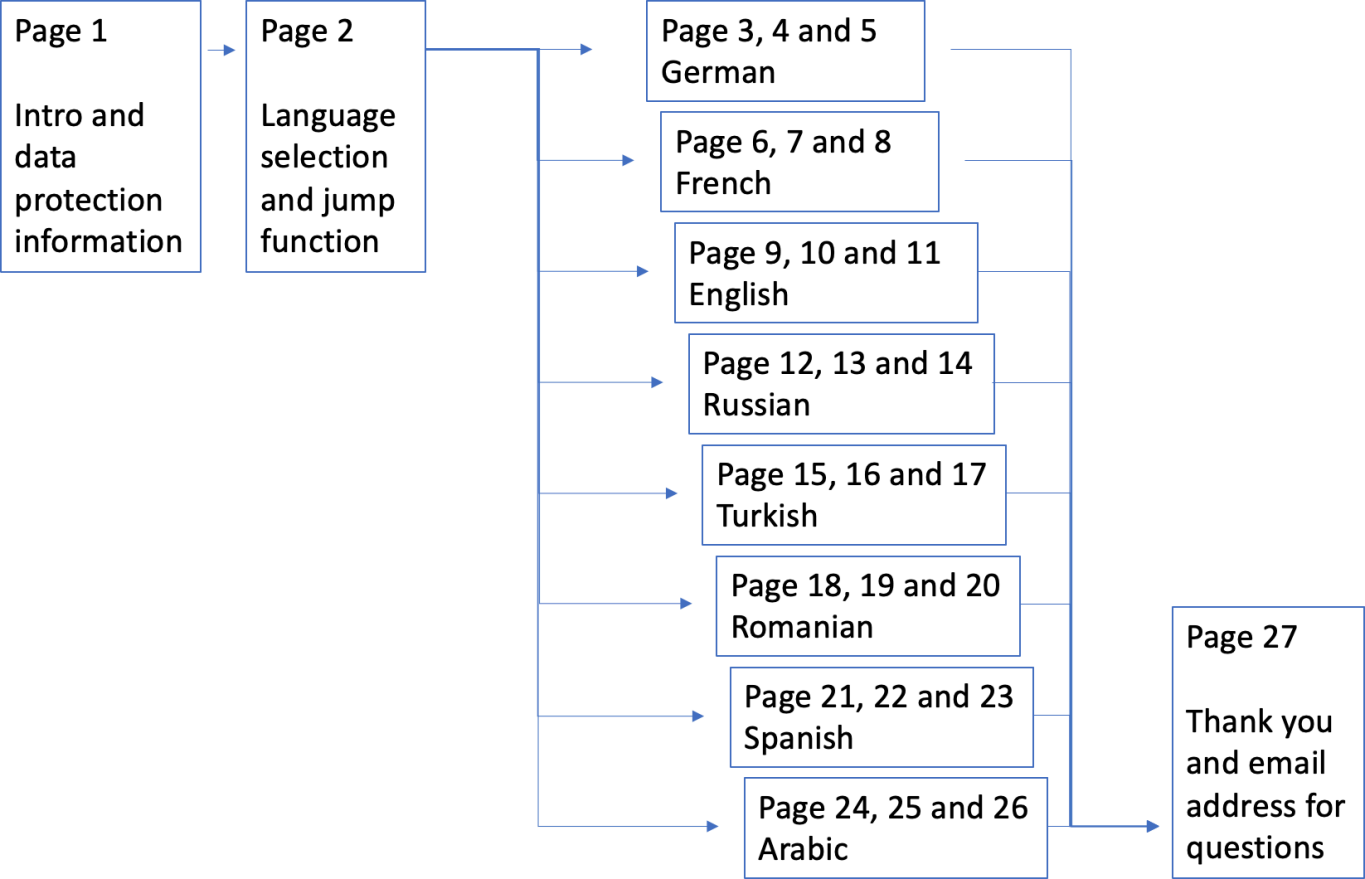
Quantitative Online-Questionnaire flow chart.

The online questionnaire included a pre-question for consent regarding the processing of collected data, a question regarding language selection, and 18 substantive questions on 3 pages as well as a final page with a thank-you note (see Figure 3). Of the 7 questions from the original paper-and-pencil questionnaire, 5 were modified and expanded to include 8 questions. A total of 10 questions were derived from the responses in the interview study.

Among the 18 substantive questions, there was an open-ended question at the beginning regarding the number of counseling sessions attended to date, as well as an open-ended question at the end for additional comments. The questionnaire also contained 1 yes-no question, 13 questions using Kunin smiley faces on a 5-point scale (see Figure 2), and 2 multiple-choice questions, the options for which were based on the categories derived from responses to the second and sixth questions of the interview study.

To ensure the anonymity of the participants, demographic data such as age and gender were not collected.

#### Data Collection

The survey was conducted between November and December 2023 using tablets within the HCS. Each participant was requested to complete the questionnaire following their counseling session, resulting in a total of 31 data entries. Due to data protection regulations, the number of individuals approached was not recorded; therefore, the response rate remains unknown.

Subsequently, from January to March 2024, the survey link was made available on the HCS’s website, a QR code was posted within the facility, and clients were informed about the survey following their counseling sessions. The number of individuals who visited the HCS during this period was also not documented for data protection reasons, resulting in an unknown response rate in this phase as well. During the period from January to March 2024, an additional 10 data entries were collected, culminating in a total of 41 evaluable data sets (with 25 aborted surveys, 38% dropouts). While response rates could not be documented due to data privacy policies, the dropout rate of 38% suggests potential limitations in sustained participant engagement, which should be considered in the interpretation of results.

#### Data Analysis

All statistical analyses of the data from the survey were carried out using SPSS version 29 (IBM). Descriptive statistics were calculated for the 13 items of the online questionnaire that were answered with a 5-point Likert scale.

#### Data Exclusion

Participants who did not consent to the data privacy statement were automatically directed to the last page and were excluded from the study. Since the survey was published on the website, there have also been instances of visits without any responses; these instances were likewise excluded from the final dataset.

## Results

### Study 1: Qualitative In-Depth Interview Study

The final sample for the interview study comprised 18 Interviews (10 clients, 6 case management participants, and 2 walk-in consultation seekers). In the context of the study, the responses to the initial question regarding the duration of engagement with counseling services exhibited a considerable degree of vagueness. Even when prompted by the interviewers, participants were unable to provide more precise answers than “for a long time,” “approximately three to five years,” and described their frequency of visits with terms like “often” and “every few months.”

The primary motivation for seeking HCS, as indicated in response to the second question, was predominantly related to issues concerning physical health. Furthermore, it was identified that the most frequently requested form of assistance was the completion of application forms, as detailed in Table 1.

**Table 1.** Overview of consulting content and types of services received

Inductively obtained categories	Mentions, n (%)	Example response
Consultancy content		
Physical health	14 (78)	“I am here because of my rheumatism.”
Mental health	13 (72)	“I have severe depression.”
Other^a^	3 (17)	“Treatment requests for my illnesses.”
Type of service		
Fill out applications and forms	17 (94)	“Well, to fill out papers for the doctor.”
Receive advice and knowledge	5 (28)	“Vaccination counselling, especially Corona.”
Referral to specialists	5 (28)	“I want to search therapy place.”, “Need to find a specialist doctor.”

a Responses could not be clearly assigned to mental or psychological health.

Responses to the third question, along with the 2 follow-up inquiries, indicated that 18 participants (100%) felt that the allocated time for HCS was adequate. In addition, 12 respondents (67%) expressed that all relevant topics could be adequately addressed during their consultations. Furthermore, 15 participants (83%) reported feeling well-supported throughout the counseling process, allowing for multiple responses.

In addressing questions 4 and 5, which pertained to the participants’ experiences with HCS and the staff, the most frequently mentioned aspects included the result-oriented and goal-oriented approach of the counselors, as well as their overall counseling attitude. Conversely, remarks regarding challenges and obstacles encountered in the counseling process were infrequently noted (see Table 2).

**Table 2.** Overview of responses on how staff and advice were experienced by clients. Multiple answers were possible.

Inductively obtained categories	Statements on the experience of consulting and employees	
	Mentions, n	Example responses
Positive atmosphere	8	“The atmosphere here is very good, quiet and pleasant.”
Result orientation and goal orientation of the consultants	34	“When I couldn’t appear in person, we then conducted over the phone.” ”When you go out afterwards, you know everything a bit more clearly.“
Counseling attitude and handling	22	“They don’t lecture me, they don’t talk down to me, they don’t tell me what to do.” ”They really treated you with respect and dignity.”
Expressed trust and sense of security	21	“I have a good feeling about the consultation.” ”I almost feel like family.”
Challenges and obstacles	7	“The consultation here is in German, I used to have it in Arabic.”

In response to the sixth question and the probing question, positive emotions were mentioned 11 times (Mentioned emotions: “a good feeling,” “I am much happier afterwards,” “I feel good”), 4 times less stress was reported (Statements about stress: “I am so nervous before,” “I go home and the stress is gone”) and 5 times answers were given that were sorted into the “Other” category (“the consultation is just good”). In response to the sixth question on emotional well-being and the associated follow-up questions, the participants registered exclusively positive statements.

When asked specifically whether and where support could be improved, the respondents provided the following insights (see Table 3): The 3 most frequently cited topics, in which the 18 participants wanted further assistance, included physical and mental health, and application processes, with each topic receiving 10 mentions (56%).

**Table 3.** Desired additional support mentioned. Multiple answers were possible.

Desired additional support	Mentions, n (%)
Referral to specialists	1 (6)
Language acquisition	2 (11)
Housing search	2 (11)
Job search	2 (11)
Networking	3 (17)
Receive case management services	5 (28)
Applications and forms	10 (56)
Physical and mental health	10 (56)

Of the 12 respondents who were either clients or walk-in customers, 5 expressed a desire for more intensive support, specifically in the form of case management services, representing 42 percent of this subgroup (28% overall).

In response to the seventh question regarding whether the participants felt they could cope independently in the future, only 2 individuals (11%) initially answered “yes.” However, both subsequently revised or qualified their answers. As a result, the majority of participants (16 persons, 89%), responded with “no.”

Among the 26 reasons mentioned were “Need for help due to chronic mental/physical health problems” (2 times), “Overwhelmed by structures in the healthcare system” (3 times), “Language barriers (eg, at the doctor’s at the doctor or hospital; mentioned 4 times),” “Barriers due to lack of reading/spelling skills” (mentioned 1 time), “No other help (caregiver, other advice center) known” (mentioned 2 times) as well as statements without further specification such as “No, I can’t cope without help” or “No way” (category “Other,” mentioned 14 times).

To obtain a statement that allows for comparability of results with other institutions in line with the Customer Loyalty Index (CLI), the final question eight asked whether clients would recommend the health care facility. All 18 participants (100%) answered affirmatively. The answers were also supplemented with statements that clients had already recommended the HCS to others or referred friends and acquaintances (5 times), that they were very satisfied (5 times) and numerous personal examples of when and how they had been helped.

In response to the probing question about what other health advice was desired, “vaccination advice” was mentioned once. In addition, the wish for a picnic was expressed once and it was said three times that it would be nice to have more time with the counselors to talk more (informal conversations). The remaining comments were praise for the interviewees and the counseling staff.

In the second probing question on the eighth question (“Is there anything else you would like to tell us?”), which was intended as a wrap-up, praise for the employees was mentioned, it was said that the advice service must not close under any circumstances, and that health and health advice is very important.

One contextual element that may have had an impact on the interviewees’ statements is the fact that the interviews were conducted on the premises of the HCS. Another contextual element could have been that the telephone interviews were conducted via the employees.

### Study 2: Quantitative Survey Study

A total of 66 surveys were started and 41 completed (dropout rate of 38%). In response to the question regarding language preference, more than half of the 41 participants chose German as their preferred language (21 participants, 51%). English and Arabic were both selected as the second most common choices, each accounting for 6 participants (15%) of the responses (see Table 4).

**Table 4.** Frequencies with which the different languages were chosen. Only 7 of the 8 languages are shown because Spanish was not chosen.

Language	Frequency, n (%)
German	21 (51)
French	2 (5)
English	6 (15)
Russian	3 (7)
Turkish	1 (2)
Romanian	2 (5)
Arabic	6 (15)

In response to the open-ended question regarding the number of previous counseling sessions, the mean of the responses was calculated to be 13.68 (SD 14.81). Regarding the second open-ended question, which inquired about any further comments or expressions of wishes to communicate, multiple participants expressed praise and gratitude for the services received.

In response to the yes or no question, one individual indicated awareness of alternative support services, specifically mentioning a caregiver. All other answers were “no” or no answer.

To investigate the subjective perceptions of the participants, items featuring various statements were employed, to which respondents could express their agreement using a 5-point Likert scale (see Table 5).

**Table 5.** Mean values and standard deviations of the responses, collected using a 5-point Likert scale.

Questionnaire items to record the subjective perceptions	Responses, mean (SD)	
I recommend the health counseling service.^a^	4.88 (0.33)	
I got help.	4.78 (0.42)	
During the conversation I understood everything.	4.65 (0.60)	
It’s easy to get an appointment.	4.61 (0.69)	
My problem was solved.	4.59 (0.61)	
There was enough time during the conversation.	4.53 (1.07)	
With the health counseling service,^a^ I now know things I did not know before.	3.94 (1.21)	
I feel comfortable at the health counseling service.^a^	3.88 (1.69)	
With the health counseling service,^a^ I can do things now that I could not do before.	3.72 (1.01)	
It is easy to get help in Germany.	3.37 (0.89)	
From now on I can help myself.	3.24 (1.20)	
I want to meet new people.	3.22 (1.47)	
I want to meet other clients of the health counseling service.^a^	2.44 (1.61)	

a The name of the health counseling service was used instead of “the health counseling service” in the web-based survey.

Regarding the 2 multiple-choice questions, which included response options based on the deductive categorical system from the interview study, the data revealed the following: the 3 most frequently mentioned sources from which individuals seeking advice had been referred were friends, unspecified others, and the employment agency (see Table 6). The primary reasons for seeking assistance from the HCS center included the desire to receive help with completing applications for authorities, having forms from and for medical practices filled out, and seeking guidance on health-related issues (see Table 7).

**Table 6.** Frequency of responses from whom consultation seekers were sent for consultation. The option “other” had a free input field in which respondents entered: “office,” “work,” and “caregiver.”

	Responses, n (%)
Other	4 (10)
Doctor	2 (5)
Debt counseling	2 (5)
Family	1 (2)
Friends	6 (15)
Job center	3 (7)

**Table 7.** Frequency of responses for reasons why the health counseling service was used. In the open entry option under “other,” the word “therapy” was entered.

Reason	Responses, n (%)
Filling out forms from doctors	11 (27)
Filling out applications for authorities	14 (34)
Advice on health issues	10 (24)
Housing search	3 (7)
Job search	6 (15)
Search for special doctors	3 (7)
Other	4 (10)

## Discussion

### Principal Findings

The qualitative study identified numerous barriers, such as language limitations, illiteracy, and psychological and physical impairments. The challenges of conducting a quantitative web-based survey for this vulnerable and hard-to-reach population could be addressed using a mixed methods approach. The most significant finding was that, contrary to previous assumptions, it was not counseling and assistance with health issues that were the primary interests of the clients, but rather support in dealing with forms and requirements imposed by institutions. It became evident that a substantial proportion of the individuals visiting the HCS suffer from multiple health problems, both physical and mental. The most significant aspect of the findings in the quantitative web-based-survey study is the implementation of the survey method and its evaluation, which enables the health care facility to continuously adapt its future work to the needs of its vulnerable and hard-to-reach clients. Only half of the respondents completed the survey in German, which clearly indicates that HCS and evaluation offered solely in German are likely insufficient. The analysis of the data further revealed that some participants required more than 30 minutes to complete the survey, as they listened to the audio recordings. This supports the assumption that the original paper-pencil assessment, which was solely in German and without explanations, was not appropriate.

A detailed examination of the qualitative study reveals that a significant portion of the work in HCS appears to focus on assisting clients with the completion of forms and documents for health insurance companies and medical professionals. This finding is not surprising, given that bureaucracy for obtaining health services in Germany has increased over recent decades, which understandably poses a significant hurdle for burdened individuals as well as those lacking sufficient system knowledge and proficiency in German. Thus, the conducted interview study highlights the substantial barriers to accessing health services and emphasizes that forms from medical professionals and health insurance companies (eg, rehabilitation requests, medical history forms, practice forms, and forms for preventive examinations) represent an overwhelming level of demand for clients. In contrast, other topics, such as education on health-promoting behaviors and vaccination advice, are rarely addressed. This may be due in part to more pressing concerns on the clients’ side (as health-related forms are often accompanied by a submission deadline) and, on the other hand, due to a lack of resources to cover additional topics.

Positively, clients reported a high level of satisfaction with the provided HCS. This could potentially be influenced by the fact that dissatisfied clients may come less frequently or seek other forms of support, or that clients may fear that the HCS will be closed if they provide negative feedback. This was explicitly mentioned in one of the interviews. At the same time, it becomes evident that clients of the health care facility with language limitations, difficulties in reading and writing, as well as cognitive and emotional barriers would be overwhelmed by the demands of the German health care system without the support provided by the facility, and would struggle to cope without the counseling.

It is noteworthy that, although the quantitative web-based survey study showed that clients are generally satisfied and many report that they learned something new and gain skills after each counseling session, they do not believe they will be able to help themselves independently in the future. It is clear that many individuals seek assistance primarily with the completion of forms and applications for medical professionals and health insurance companies, and they perceive this task as overwhelming in the long run. Bureaucracy within the health care system thus presents a significant barrier that they do not believe they can ever manage alone.

Other HCS (individual counseling, information and education, networking, organization of self-help groups, and health education through lectures and workshops), which would also provide substantial benefits to these individuals, were, however, not requested. At the same time, the data indicate that very few participants were referred by doctors and authorities. This could be attributed to a lack of awareness regarding the number of people who need assistance with form completion or to inadequate knowledge among medical professionals and authorities about the availability of HCS that offer such support. There is an urgent need for increased awareness in this regard.

Both studies may be limited by the small number of participants and the specific selection of clients visiting the HCS center, which may not necessarily allow for broader generalizations to the entire population.

Furthermore, biased self-reports from clients (eg, due to psychological stress) could have affected the validity of the data collected. In the first study he opinions of walk-in clients are likely underrepresented, as only 2 out of 18 respondents (11%) in the interview study sample belonged to this demographic, while staff at the HCS center estimate that approximately a quarter of consultations involve walk-in clients. The distribution of responses among clients, cases, and walk-in clients in the web-based survey is also unclear. Furthermore, it cannot be excluded that some individuals participated in both the interview study and the web-based survey, which would reduce the generalizability of the findings. Also, it cannot be guaranteed that the audio content was always understood, both auditorily and cognitively, by the participants who chose to have the questions read aloud to them instead of reading them themselves. In addition to the previously described challenges of digital accessibility for this group, limitations due to psychological distress and the difficulty of reaching this client population may also play a role. However, the relatively small sample does represent the majority of the current clients of the health care facility in terms of their challenges.

In conclusion, the findings of this study clearly indicate that support with administrative requirements is of paramount importance for clients within the German health care system. The overwhelming bureaucratic hurdles and the associated burdens underscore the need for reforming health services to ensure that clients not only receive the necessary medical assistance but also the support needed to successfully navigate the complex health care landscape. These insights carry broader implications for the design of health services, which should be more inclusive and accessible to better address the needs of these vulnerable groups. These insights are of great significance, as they underscore for a broader awareness of the challenges faced by clients. Furthermore, a training program for staff on completing applications for medical services is now being considered. Ultimately, however, health services must be designed to be more inclusive and accessible to enable the needs of the entire population to be met without intensive support structures.

### Comparison With Previous Work

The findings of this study are consistent with a variety of research concerning medically underserved populations.

Once again, it was shown that in-depth interview are very well suited to identifying client experience with the health system [24]. Participants in the qualitative in-depth interview study reported that Arabic language counseling services were previously available; however, these services have been discontinued due to a shortage of staff. The issues of inadequate expertise and personnel shortages in HCS have also been documented in previous studies [1,2,28].

The web-based survey indicates that collecting data on health uptake among non-native speakers poses significant challenges [29] and that it is important to involve several stakeholders from the outset and to pay attention to user-friendliness [30]. Furthermore, it has been confirmed that this demographic faces specific barriers, of which healthcare providers and authorities are only partially aware [31]. The notably lower data collection rates during the second period of the study—where data were gathered through a link and QR code instead of in-person consultations with tablets—alongside a high dropout rate (n= 25, 37%), suggest that individuals seeking HCS encounter difficulties in navigating digital media. This observation is also supported by previous research [11,15,23].

Overall, the results confirm that the low demand for health services in Germany, as evidenced in earlier studies [2,4-7], may indeed be attributed to the challenges faced by particularly burdened individuals, specifically those who are in greatest need of assistance [31].

### Conclusions

In summary, the results of the 2 studies underscore the complex challenges faced by individuals seeking assistance at HCS centers, particularly in relation to the completion of forms required by health care professionals and insurance providers. The qualitative in-depth interviews reveal that a significant proportion of clients are overwhelmed by the bureaucratic demands of the health care system, which detracts from opportunities for health promotion and education. The quantitative analysis further suggests that language barriers and insufficient referral practices limit access to resources, highlighting the inadequacy of German-only evaluation form. While clients express satisfaction with the services received, they largely lack confidence in their ability to navigate the system independently in the future. These findings indicate an urgent need for enhanced awareness among health care providers and authorities regarding the challenges faced by medically underserved populations, as well as the importance of effective networking and support systems to facilitate holistic patient care.

## Abbreviations

AI: artificial intelligence
CLI: Customer Loyalty Index
HCS: health counseling service
